# Time-Series Analysis of Embodied Interaction: Movement Variability and Complexity Matching As Dyadic Properties

**DOI:** 10.3389/fpsyg.2016.01940

**Published:** 2016-12-12

**Authors:** Leonardo Zapata-Fonseca, Dobromir Dotov, Ruben Fossion, Tom Froese

**Affiliations:** ^1^Plan de Estudios Combinados en Medicina (MD/PhD), Facultad de Medicina, Universidad Nacional Autónoma de MéxicoMexico City, Mexico; ^2^Centro de Ciencias de la Complejidad, Universidad Nacional Autónoma de MéxicoMexico City, Mexico; ^3^Departamento de Estructura de la Materia, Instituto de Ciencias Nucleares, Universidad Nacional Autónoma de MéxicoMexico City, Mexico; ^4^Departamento de Ciencias de la Computación, Instituto de Investigaciones en Matemáticas Aplicadas y en Sistemas, Universidad Nacional Autónoma de MéxicoMexico City, Mexico

**Keywords:** time-series analysis, social interaction, social cognition, embodied cognition, enactive, complexity

## Abstract

There is a growing consensus that a fuller understanding of social cognition depends on more systematic studies of real-time social interaction. Such studies require methods that can deal with the complex dynamics taking place at multiple interdependent temporal and spatial scales, spanning sub-personal, personal, and dyadic levels of analysis. We demonstrate the value of adopting an extended multi-scale approach by re-analyzing movement time-series generated in a study of embodied dyadic interaction in a minimal virtual reality environment (a perceptual crossing experiment). Reduced movement variability revealed an interdependence between social awareness and social coordination that cannot be accounted for by either subjective or objective factors alone: it picks out interactions in which subjective and objective conditions are convergent (i.e., elevated coordination is perceived as clearly social, and impaired coordination is perceived as socially ambiguous). This finding is consistent with the claim that interpersonal interaction can be partially constitutive of direct social perception. Clustering statistics (Allan Factor) of salient events revealed fractal scaling. Complexity matching defined as the similarity between these scaling laws was significantly more pronounced in pairs of participants as compared to surrogate dyads. This further highlights the multi-scale and distributed character of social interaction and extends previous complexity matching results from dyadic conversation to non-verbal social interaction dynamics. Trials with successful joint interaction were also associated with an increase in local coordination. Consequently, a local coordination pattern emerges on the background of complex dyadic interactions in the PCE task and makes joint successful performance possible.

## Introduction

Recently cognitive science has started to adopt a multi-scale and dynamical systems account for different aspects of human behavior (Dumas et al., 2014). Complexity sciences are a formal way for adopting such an approach, as they have the advantage of studying a wide variety of phenomena from both holistic and dynamic perspectives. This way of thinking has yielded many insights into the behavior and underlying patterns of many processes in different fields like sociology, economics, medicine, biology, and cognitive sciences (Gershenson and Heylighen, 2005).

Compatible with complexity sciences is the enactive approach to cognition, which is based upon two main ideas. Firstly, the mind is considered as embodied so it cannot be reduced to the brain activity, and similarly, the body cannot be only regarded as a sensorimotor reservoir (Thompson, 2007). Via our embodiment we are intentionally directed toward and entangled with the world. Secondly, the lived experience of the cognitive agent, which is embedded into an environment, influences its actions (Froese and Di Paolo, 2011; Colombetti, 2014).

Therefore, the interactions between environment, body and brain become relevant to the understanding of the mind and thus, the components involved in human cognition cannot be studied as isolated entities, but rather as a whole dynamical system (Beer, 2000). This dynamic approach can be generalized from the individual to the dyadic situation such that social interaction is considered to be a relational property of the whole embodied and situated two-agent system (Froese et al., 2013b).

A way of studying social cognition from a dyadic, dynamical and embodied perspective is the perceptual crossing experiment (PCE), originally introduced by Auvray et al. (2009). In this paradigm, pairs of participants are situated in an invisible one-dimensional and periodic virtual space (i.e., a loop) in which they move their avatars. Participants make haptic contact with three types of objects when they pass over them in the virtual space. Two of these, slightly spaced apart, constitute the partner’s avatar and its ‘shadow’; only the avatar also coincides with the partner’s contact sensor and thereby enables responsive interaction. The remaining object is a static decoy which does not change its position over time.

The PCE has proved to be a successful paradigm for considering embodiment and the relevance of the interactions that take place in social cognitive tasks (Rohde, 2010; Auvray and Rohde, 2012). It has also been able to take into account the subjective experience of the participants as well as support the idea that in some cases social cognition of an individual is constituted by its social interaction with others (De Jaegher et al., 2010; Froese et al., 2014a).

Consequently, the interaction itself has become a process thoroughly studied by the PCE and the study of it has supported the development of new theories of social cognition like second-person neuroscience, in which the interaction dynamics between two engaged subjects are of the utmost importance (Schilbach et al., 2013).

Recent work by Bedia et al. (2014) have pointed out that existing analyses of the PCE, both in the simulation and the human behavioral scenarios, have implicitly assumed that the emergence of social movement phenomena can be reduced to a single and relatively short time scale, thereby neglecting potentially the multi-scale organization of interaction dynamics constituted by long-range correlations and other fractal phenomena. Indeed the human behavior has been formally studied from a systemic point of view. Particularly, social interaction has been regarded as a process to which some dynamical systems tools can be applied (Froese et al., 2013a). So it is sensible to focus on the coordination among multiple temporal scales, rather than on isolated scales or even on a single one (Ihlen and Vereijken, 2013).

In this way, systems biology and complexity sciences become pertinent because they focus on the interactions rather than on the individual components of any given system. Particularly, time-series analysis (a tool used for studying complex systems, e.g., Hardstone et al., 2012; Fossion et al., 2013) allows a dynamical assessment of the interactions underlying a system’s behavior. Furthermore, this approach provides high temporal resolution, as well as holistic and multi-scale accounts of such systemic interactions (Fossion and Zapata-Fonseca, 2015).

In the context of embodied social interaction, the slower time scales involving conversation and body movements have been shown to mediate important parts of the social exchange (Schmidt et al., 2014). Bedia et al. (2014) confirmed the multi-scale character of human–human interaction in a modified and constrained version of the PCE.

Taking into account the criticism by Bedia et al. (2014) and appealing to the pervasiveness of scaling laws in the human behavior (Kello et al., 2010), we have adopted a time-series analysis perspective for studying embodied social interaction. These analytical tools permitted the consideration of the dynamics occurring over the full length of the trials in the PCE study carried out by Froese et al. (2014a). Besides, it was possible to measure both the individual and dyadic levels of embodied dyadic social interaction and to account for the presumed presence of scaling properties.

The frequency and variability domains were assessed by means of the standard Fourier spectral analysis, yielding different results according to different qualities of social interaction, both at the objective and subjective levels.

The scaling domain’s assessment was done by computing multiple Allan factor coefficients within each time-series. Such method was chosen due to the nature of the signals obtained from the current experiment (further details in Section “Allan Factor Analysis for Temporal Clustering”). Afterward we calculated the complexity matching between the Allan factor coefficients of individuals conforming a pair. Complexity matching is a particular case of distributional matching (DM), but it is important to notice that complexity matching is rather a general method in the sense that it can be computed using correlation coefficients, DFA exponents, or Allan factor values, as it was the case in this research. Complexity matching was carried out in order to test out the theoretical hypothesis that recognizes embodied interaction as a multi-scale phenomenon.

Importantly, it is possible that complexity matching of binary event series is a trivial consequence of local coordination and of the multi-scale dynamics exhibited at the individual level. In particular, if the dyad members somehow become synchronized, irrespective of the complex motor behavior exhibited at the level of the individual, then complexity matching will necessarily follow from the synchronization. This would greatly weaken the potential evidence that complex multi-scale interactions support dyadic embodied coordination in the PCE.

To this end we quantified local coordination using methods that are only sensitive to direct local coordination. Two different methods were used because there is a lack of an *a priori* understanding of how to operationalize local coordination in the PCE task which has a special and novel character: (1) the cross-correlation between the series of movement onset-offset intervals of both partners; and (2) an event synchronization metric developed for neural spike trains. The latter, albeit used outside its traditional domain, is directly applicable because the movement onset-offset events constitute a binary temporal representation of the motor behavior (details can be found in the Supplementary Material). These events are a relevant dimension of coordination because the ideal way to perceive the presence of a human avatar at a given moment in the PCE setup is when the perceiver is not moving while the avatar is moving in the vicinity. So coordination of periods of movement and no movement between partners facilitates the successful performance of the task.

The upshot of our analyses is that applying time–series analysis to embodied dyadic social interaction allows investigating the whole dynamics of the different modalities of social interaction observed in the PCE. Moreover, different scales of temporal resolution intrinsic to social phenomena can be studied when adopting the approach here proposed perspective. Particularly, our research suggests that due to the nature of the task dynamics, important information about movement coordination can be obtained by considering the PCE as a point process instead of as continuously fluctuating signals. Consequently, methods for binary event-based series, such as the Allan factor, are required.

Remarkably, our work showed that the time-series from the PCE and those from dyadic conversations share certain properties. Specifically, our findings are compatible with previous evidence in which complexity matching proved to be a suitable tool for measuring higher-order complex coordination coupling at the dyadic level (Abney et al., 2014).

Furthermore our results lead us to conclude that time-series analysis, in the context of embodied dyadic social interaction, is a potential tool for understanding how the on-line social interaction is modulated by different environmental contingencies, as well as by individuals’ and collective behavioral dynamics (De Jaegher et al., 2010; Froese and Di Paolo, 2011; Schilbach, 2016). Finally, this line of research could yield some light into the search of behavioral markers of social interaction impairments by means of time-series analysis (Fitzpatrick et al., 2016).

## Materials and Methods

### Minimal Social Cognition Experiment

The participants of the PCE that we analyzed (Froese et al., 2014a) were healthy volunteers recruited from acquaintances at the University of Tokyo (*N* = 34). There were 25 from Japan and the rest were from diverse nations. Six were female. The mean age was 29 years. The Ethical Committee of the University of Tokyo approved the study. All of the participants gave their written informed consent before taking part in the study. Readers already familiar with the PCE can skip ahead to Section “Time-Series of Player’s Instantaneous Velocities.”

**FIGURE 1:**
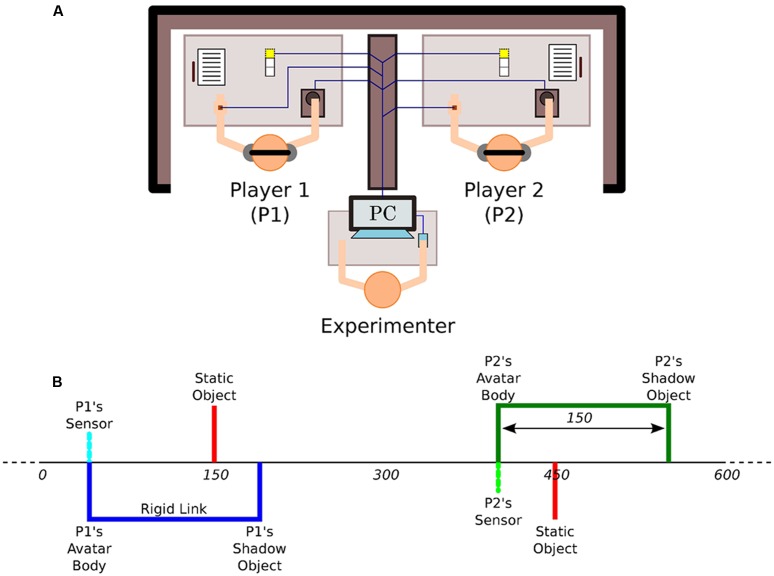
**Experimental set up. (A)** The participants are placed in separate locations and can only interact with each other by means of a minimal haptic human–computer interface that provides all-or-nothing vibratory feedback depending on whether a player’s avatar overlaps with another virtual object or not. **(B)** The virtual space consists of an invisible 1D circle that wraps over after 600 units of space and in which players can encounter three different objects of equal size: the other’s avatar, the other’s shadow, and a static object. Adapted from Froese et al. (2014a).

The PCE is considered a minimal social cognition paradigm because pairs of humans have to recognize and socially interact with each other within a very constrained environment (Auvray and Rohde, 2012). The variation of this paradigm employed by Froese et al. (2014a) consists of an invisible one-dimensional virtual space of 600 pixels width with connected endpoints, thus forming a 1D loop, into which two participants aim to encounter each other’s avatars and establish an enduring interaction. Each trial lasts for 60 s and 15 trials were performed by each dyad. Additionally, each player can encounter two different equally sized objects other than her partner in the virtual shared environment, namely a fixed object with a constant position and the so-called shadow or lure object that moves exactly as the partner but lagged in position. The interaction in this experimental set-up occurs by means of haptic feedback and active movements throughout the horizontal axis. The movement in the virtual space was mediated by a trackball controlled by the dominant hand of the participants and the haptic feedback by a vibrating device located in the non-dominant hand. The haptic feedback was discrete in nature and enabled when the sensor crossed any of the three objects (static decoy, avatar of the partner, and shadow avatar of the partner) in the virtual space (**Figure 1A**). Such feedback consists of a vibration with both fixed intensity and duration regardless of the object that is encountered. Participants were instructed to click (at most once per trial) if they felt that they had found the other player. This experimental setup was carried out on a team-based competitive tournament basis so that the players were encouraged to solve the task collectively. The winner dyad was the one that more accurately performed in the each other’s recognition task measured by clicks accuracy.

From such a set-up, it follows that the strongest interaction happens when both players’ sensors are turned on simultaneously, and consequently both participants feel a vibration at the same time (**Figure 1B**). In contrast, participant A feels a vibration when Pa-sensor crosses Pb-shadow but participant B does not receive any feedback. Participant A would also feel a vibration when crossing the Pa-static object. The difference here with respect to the previous two scenarios is that the static object would always be there, it would not escape if one crosses it and then returns to it. If one crosses either Pb-sensor or Pb-shadow, however, one might not find them at the same place when going back. These two virtual objects always move in an identical manner, but only Pb-sensor can be responsive to contact. Participants are assigned static objects individually and uniquely. This means that Pa-static and Pb-static are at different locations and can only be felt by their respective assigned member of the dyad. Given that all three types of objects are equal in size, the only way for recognizing the partner as such the participant must rely on the dynamics of the sensorimotor loop that emerges out of the interaction itself.

In order to quantify the success of the interaction, Froese et al. (2014a) assessed the performance from both objective and subjective perspectives. The experimenters asked the players to make a click per trial whenever they considered that an interaction with another human was taking place. The objectivity of the social encounter was measured by the accuracy of the clicks that the players made. Importantly, the rate of click accuracy was not disclosed to the participants until the end of the whole experiment. Thus, the objective evaluation was an external and detached one.

The subjective assessment was performed by means of the participants’ reports at the end of each of the 15 trials. This first-person report was based upon a Likert scale called Perceptual Awareness Scale (PAS), originally proposed by Ramsøy and Overgaard (2004) for the study of visual awareness. The scale was adopted by Froese et al. (2014a) for the study of social awareness assigning different values depending on the clarity of the other’s presence that the participants experienced. The PAS was scored as follows: 1, no experience; 2, vague impression; 3, almost clear experience; and 4, clear experience.

### Time-Series of Player’s Instantaneous Velocities

Originally, 510 time-series were obtained corresponding to the 15 trials made per participant in the PCE carried out by Froese et al. (2014a). As mentioned before each trial lasted 60 s and because of the 100 ms sampling rate the time-series length was approximately of 5980 points. Such points represent the sequence of a player’s positions while exploring the shared virtual space where the corresponding partner was also moving, that is, their behavioral trajectories.

**FIGURE 2:**
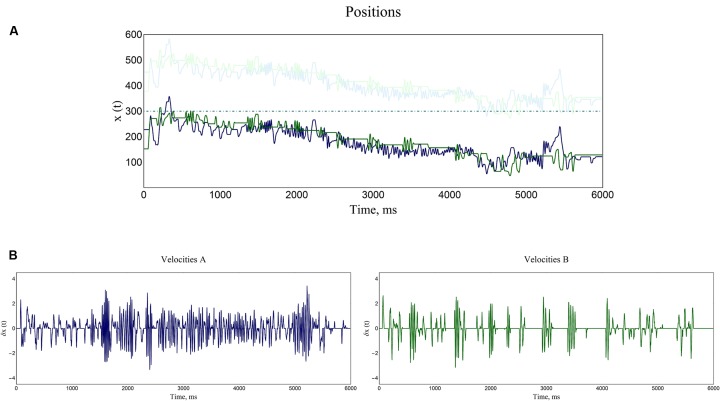
**Time-series of a representative trial. (A)** Both participants’ positions are plotted, as well as the positions of the objects which they could encounter with (shadow objects and static objects). In this example an interactive turn-taking of movements is taking place, and a jointly drifting through the virtual space is conspicuous. **(B)** The instantaneous velocities (derivative of the players’ positions) are shown for both participants of the dyad. Notice that all the values oscillate around zero, meaning that there is no trend in this new time-series.

It is worth mentioning that the PCE space and the trackball movements entail a periodic domain, i.e., the axis on which the players move wraps over in a 1D circle fashion. Due to the bounded periodic domain of the PCE space, the state trajectories of positions could exhibit discontinuous jumps. These abrupt changes were corrected using a standard unwrapping algorithm for phase angle data. In **Figure 2** an example of a trial is shown.

As in Bedia et al. (2014) we converted these sequences of position measurements into time-series of velocity. The instantaneous velocity (rate of change of positions) of each player was calculated by subtracting the successive positions for every point of the time-series and dividing by 0.01, the sampling interval in seconds; so this procedure yielded the derivative of position with respect to time (*dx/dt*).

The velocities were preferred to the positions because velocity is directly related to players’ behaviors as a surrogate of the track ball movements. Moreover, players did not have access to their own position information so they were aware only of their changes in positions. So the velocity was what they were able to control. It is important to mention that the positions exhibited strong recurrent long-range trends. This has been identified as a source of spurious results in the sort of scaling analysis considered here (Bryce and Sprague, 2012). Fluctuations in velocities, on the other hand, are centered on a constant mean of zero velocity entailing a lack of trend.

### Fourier Spectral Analysis

For this analysis we identified features of specific types of social interaction by studying the cases in which subjects reported having had a real experience, i.e., subjects whose PAS score was equal to one, meaning no experience at all, were excluded. Furthermore, PAS = 2 and PAS = 3 were grouped together because both items referred to an ambiguous experience (AE), in contrast to the clear experience (CE) of PAS = 4. This arrangement yielded consequently two situations (“ambiguous” and “clear experience”) for the subjective measure (SM).

It is also worth considering the comparison between trials in which both players clicked correctly (“joint success,” JS) and those in which there was at least one click response but either one or none of the players clicked correctly (“individual response,” IR). Two groups were identified according to this objective measure (OM): players whose clicking was indicative of collaboration and players who were less well coordinated with their partner but who still produced a response. The second group is indicative of a judgment regarding the partner’s presence which was made under conditions of less effective coordination.

**FIGURE 3:**
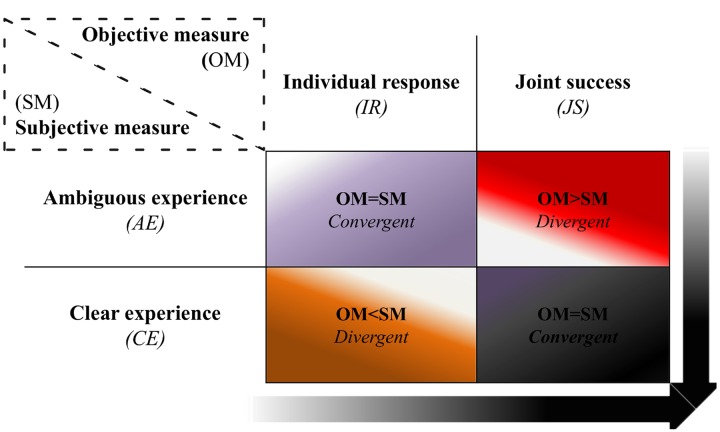
**Classification of social interaction.** The black cell represents the most social way of interacting, in which both players succeeded in making a correct click and also reported having had a clear experience of each other. In contrast, the opposite cell (purple) stands for a less social way of interacting, since the objective and subjective evaluations show that encounters were uncoordinated (individual response only) and ambiguous. The sample sizes for each cell are referred in the main text. The arrows correspond to the gradient toward which the quality of interaction moves from less to more social interaction, being optimal in the lower right corner. OM, objective measure; SM, subjective measure; IR, individual response; JS, joint success; AE, ambiguous experience; CE, clear experience.

Thus, four groups were assessed with this method, namely: IR and AE (*n* = 87); JS and AE (*n* = 135), IR and CE (*n* = 28); JS and CE (*n* = 115). Taking into account such classification we identified two general types of situations depicted in **Figure 3**:

(1) Convergent: Both OM and SM of social interaction matched with each other (purple and black cells); and
(2) Divergent: OM did not match with the SM (red and orange cells).

The standard Fourier spectral analysis was made for 365 time-series of individuals’ velocities after excluding 145 time-series belonging to participants whose performance were not useful for the present study according to the criteria described above (no click at all or a click with PAS score of 1).

The Fourier transform was applied in order to assess the frequency domain and to get a general notion of the signals’ structure. As there was no trace of any power-law distribution regarding the frequencies, scaling methods were not considered in this first evaluation.

As a common practice, when assessing a time-series, the first step is to compute the Fourier power spectra. Accordingly, in the current study we firstly applied the Fourier transform to the individuals’ time-series of instantaneous velocities.

The Fourier transform is a linear transformation that decomposes a discrete time-series *x (n)* = *x_1_*, *x_2_*, … *x_N_* of *N* successive observations as the sum of periodic basis functions equation (Butz, 2006),

$$x\left(n\right)=\overset{N-1}{\underset{k=0}{\Sigma }}X_{k}e^{{i\omega }_{k}n}$$

where *X_k_* are complex Fourier coefficients that serves as weights for the periodic functions,

$$X_{k}=\frac{1}{N}\overset{N}{\underset{n=1}{\Sigma }}x\left(n\right)e^{-{i\omega }_{k}n}$$

where the frequency ω*_k_* = ± 2π*k*/*N* indicates *k* complete cycles over the whole duration of *N* data points and where frequencies can be positive and negative. The so-called Fourier power spectrum,

$$P(K)=\{{\left|X_{k}\right|}^{2},k=0,...,N-1\}$$

is then the collection of the power |*X_k_*|^2^ contained in each of the periodic basis functions and where the powers are ordered according to frequency *ω_k_*.

It is worth to mention that the variability of the time-series can also be studied when applying such transformation due to the fact the power |*X_k_*|^2^ can be interpreted as a partial variance. Specifically, Parseval’s theorem establishes that cumulative sum over all partial variances equals the total variance (Var) of the original time-series,

Finally, the Nyquist theorem establishes that the maximal frequency is ω*_k_* = ± 2π*(N*/2*)*, i.e., *N*/2 complete cycles over the whole duration of the time-series with length *N*, or 1 cycle every 2 data points (Butz, 2006). Therefore, the power spectra are usually plotted for half of the length of the time-series, as it is symmetrical when plotting all the frequencies without neglecting the sign. More information about power spectral analysis with the Fourier transform can be found in the “*Fourier Spectral Analysis*” of the Supplementary Material.

The main advantage of Fourier spectral analysis is that the single value of the total variance Var of the time-series is decomposed in contributions at different frequencies, which allows distinguishing between time-series with equal Var but dominant contributions from different frequency ranges.

Additionally, the power spectrum can also be used for assessing power-law distributions that might be presence in a given time-series, which would mean that low frequencies or slow waves contribute more to the series than high frequencies or rapid waves (Muñoz-Diosdado et al., 2005). Such a behavior is formalized by the relation P(*k*) ∝ 1/*k*^β^, where the scaling exponent *β* is the slope of the fitted line in the double logarithmic plot of the power spectra.

### Distribution of Movement and Non-movement Intervals

**FIGURE 4:**
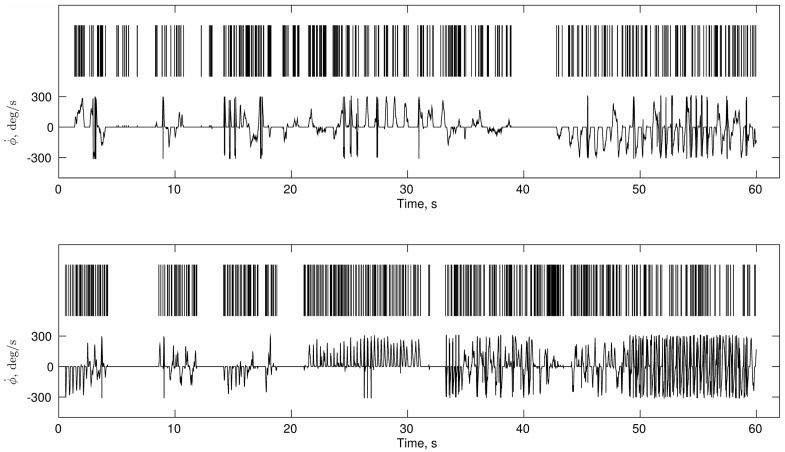
**Continuous and discrete representations motor activity.** Two partners’ velocities from a sample trial are shown in the lower half of each panel. The upper series are the corresponding point processes obtained from the initial and terminal points of zero-acceleration.

Proper selection of analytical tools requires taking into account not only the theoretical hypothesis but also the properties of the signal that is being analyzed. In the present experiment, the beginnings and ends of activity make a salient feature of the perceptual crossing task dynamics. **Figure 4** suggests that participants did not move continuously but made frequent stops during which they were “silent.” Supplementary Figure S1 shows the distribution of such “silences” which reveals a wide range of silent periods. Note that they appear to obey the same scaling law as the distribution of movement periods (see IEI Power-Law Distribution).

On average 42% of the velocity time-series across all trials and participants consisted of silences that lasted ten or more consecutive samples (100 ms or more). This means that nearly half of each trial is not singular and would greatly bias analyses such as DFA that assume everywhere-singular behavior (see Supplementary Material for more in-depth discussion).

This behavioral pattern can be seen preferentially in terms of binary series of onsets (events) and endings of activity, i.e., as a point process (see **Figure 4**), in which the inter-event intervals (IEI) become more relevant than the fluctuations of the data points.

This type of mixture between continuous and discrete motor activity in the PCE and the distribution of the intervals led us to adopt an analysis of Poisson-like processes. Accordingly, a point process of zeros and ones (see **Figure 4**) was obtained for each trial and individual by taking the first and last points of zero acceleration in each bout of constant velocity (zero acceleration). Then the distribution of the intervals of acceleration onsets and offsets (IEI) was computed as a preliminary step before applying the scaling analysis that we describe in Sections “Complexity Matching” and “Local Coordination.”

### Complexity Matching

The motor behavior in the PCE task is reminiscent of speech time-series in the sense that both exhibit clustering of discrete events (movement or speech onsets) on multiple time scales.

Recently, a study on dyadic conversations showed that the scaling relation of clustering in acoustic events (speech onsets) taken as a point process reveals important information about the dynamics of conversational speech (Abney et al., 2014).

Particularly, the authors reported that speech in conversation exhibits a power-law or heavy-tail distribution over the available time scales of activity. Also, they found evidence for complexity matching, meaning that the power-laws characterizing two partners’ speech tended to converge when they were engaged, especially when in an affiliative conversational style.

Complexity matching is considered to be a special case of so-called DM and theoretically is thought to be a form of higher-order coordination related to the maximization of information exchange between coupled complex systems (West et al., 2008).

It is important to mention that complexity matching is the multi-scale systemic variation of behavioral matching (BM) which has been used to refer to coupling phenomena like alignment, entrainment, convergence, and synchronization (Louwerse et al., 2012; Abney et al., 2014).

We quantified the complexity matching for the whole data set (*N* = 510) corresponding to every trial *(N* = 15) for every member of the dual teams (*N* = 34). This decision is also supported by the fact that the aim of this part of our research was to have a comprehensive understanding of the embodied interaction as a whole instead of trying to classify the phenomenon in terms of its success as we did in the power spectra analysis.

#### Allan Factor Analysis for Temporal Clustering

Following Abney et al. (2014), we computed the complexity matching on the basis of the individual Allan factor coefficients of dyad members in each trial. Allan factor is a form of variance for point processes. Consequently, complexity matching compares the power-law clustering in two point processes by comparing their scaling functions.

For a given point-process, the Allan factor variance A *(T)* is obtained as follows:

• The signal is segmented into *M* adjacent windows of size *T*, determined by the number of non-overlapping windows covering the time-series.
• The number of events N_j_ is counted within each window *M*. The number of events are indexed by *j* = 1 to *j* = *M*.
• Afterward, a ratio similar to a coefficient of variation is obtained. The expected value of the squared differences d(T) = N_j+1_(T) - N_j_(T) in counts between adjacent windows of a given size *T* is normalized by the mean counts of events per window,

The A (*T*) for timescale *T* is given by:

$$A(T)=\frac{\langle {d(T)}^{2}\rangle }{2\langle N(T)\rangle }$$

The relation *A*(*T*) ∼*T^α^* indicates a Poisson process when *α* = 0, i.e., *A*(*T*)∼1 for all *T*, whereas if *α* > 0 it means that the distribution of the clustering follows a different power-law.

In the present study, the number of windows ranged from 2^12^ to 2^3^ which resulted in a minimum window size *T* of 14.6 ms and maximum of 7.50 s. In the Section “Movement Invariants in the Frequency and Time Domains Classify Social Interaction” we suggest the kinds of motor behavior that might be included within these temporal scales.

To determine complexity matching between two participants in a given trial, a distribution similarity index is calculated by comparing the respective Allan factor functions,

$$D_{a,b}=-\underset{T}{\Sigma \,}\log\left|A\left(T_{a}\right)-A\left(T_{b}\right)\right|$$

### Local Coordination

#### Cross-Correlation

As suggested by Abney et al. (2014), BM in the form of local coordination can be studied by the cross-correlation. Consequently, the cross-correlation analysis of the movement durations was computed for all the 510 trials like in the complexity matching analysis, as the main purpose was to assess the embodied dyadic interaction from a more general perspective. Movement duration is defined as the time between motion onset and subsequent end of motion so that the events can take only two values. From each trial time-series of an individual series of movement durations was obtained. This is passed through a cross-correlation with the partner’s series. Cross-correlation is an iteratively repeated correlation where the one series is lagged relative to the other. We used the peak positive correlation between the movement durations of the two partners for each trial, as well as the lowest negative correlation (negative peak). These were analyzed separately as two separate dependent variables.

#### Event Synchronization as Spike-Train Synchronization

Due to the binary character of the events series, the most direct way to quantify local coordination is to use measures developed for detecting synchronization among neural spike trains (see Supplementary Figure S2). The SPIKE-distance measure (Kreuz et al., 2013), which is based and improves on the former ISI-distance method (Kreuz et al., 2007), addresses several important issues associated with correlation-based measures of spike train synchronization. It is scale-independent, parameter-free, and sensitive to individual synchrony events even when they are mixed with many non-synchronous events. The details of the algorithm are described in the Supplementary Material. Note that the sort of local coordination where the events in the one series are matched in time by the events in the other series implies complexity matching but the reverse is not necessarily true. For this reason, SPIKE-distance is appropriate for testing whether local coordination of complex individual behavior is what drives dyadic complexity matching.

### Statistical Analysis

#### Linear Mixed-Effects Modeling

In the current experiment, subjective experience (PAS scores) and objective performance (either presence or absence of JS) are trial-by-trial covariates of motor behavior that hypothetically are associated with it. In order to account simultaneously for this association while also controlling for a potential practice (trial) effect, we employed linear mixed-effects modeling (LMEM), a technique developed for the statistical analysis of longitudinal studies with multilevel designs (Singer and Willett, 2003). It resembles the regression of an outcome variable against multiple predictors but can also deal simultaneously with predictors at different levels, i.e., time-varying predictors as well as constant randomly assigned grouping factors such as participant identity. It treats each participant’s trials as a trajectory of successive observations and uses dummy variables as trial-varying predictors to represent the *Performance (Per)* and *Experience (Exp)* observed in the respective trials. The model template consisting of all predictors and two-way interactions is

$$\begin{array}{c} Y_{ij}=\beta_{00}+\sigma_{0i}+\beta_{10}{Trial}_{ij}+\beta_{20}{Exp}_{ij}+\beta_{30}{Per}_{ij}\\ +\beta_{40}{Exp}_{ij}{Trial}_{ij}+\beta_{50}{Per}_{ij}{Trial}_{ij}+\beta_{60}{Exp}_{ij}{Per}_{ij}+\sigma_{ij} \end{array}$$

This combines the IR trajectories into *Y*, a 17 × 15 (dyads*trials) matrix. The predictors are also matrices of the same size. For example, *Trial* is a 17 × 15 matrix which has identical rows growing from zero to 14.

To facilitate the interpretation of the fitted models, consider that when a given predictor is time varying but binary, as is the case with *Experience* (clear or ambiguous) and *Performance* (JS or IR), its effect is to impose a constant shift in the outcome variable in a trial-dependent manner. The magnitude of this shift is estimated by the coefficients for the respective predictors. Maximum-likelihood-estimated LMEM coefficients are comparable (but not equal) to regression slopes and intercepts estimated using least-squared-error. For example, a coefficient of β_30_ estimated for *JS* trials means that trials where *JS* was observed tended to be β_30_ higher on the outcome variable than *IR*, regardless of their order. A coefficient of β_50_ for the interaction between *Trial* and *Per* means that each subsequent trial where *JS* was observed tended to be β_50_ higher than the previous trial where *JS* was observed.

The relevant variables (total power, cross-correlation of IEI, SPIKE-distance) were fitted independently against *Trial*, *Exp*, and *Per* using a recommended model development procedure (Singer and Willett, 2003) in *lme4*, a dedicated statistical package for *R* (Bates et al., 2015). The procedure consists of incrementally including the predictors and their interactions in a sequence of models and evaluating the increase in goodness-of-fit associated with each expanded model. A thorough explanation of this modeling can be found in the Supplementary Material.

#### Surrogate Dyads

For stronger statistical confirmation of complexity matching, a test is performed by constructing surrogate dyads comprised of the signals from participants who did not interact and then comparing the *D_a,b_^Original^* and *D_a,b_^Surrogate^* values using a *t*-test. For the surrogate test, a large sample of fake dyad trials was produced by coupling the point process from each trial and each participant with a point process, randomly selected between the two members of a dyad, from each other trial of each other dyad. This means that each point process was paired with half (240) of all possible point processes produced by members of other dyads. The resulting two samples of *D_a,b_* (for each member of the dyad) were pooled and averaged to obtain a surrogate *D_a,b_* for the given trial. These were further averaged to obtain a single *D_a,b_^Surrogate^* per dyad to be compared to the *D_a,b_^Original^* for the dyad. This procedure was based on the one described by Abney et al. (2014).

The same surrogate analysis was applied to the cross-correlation and SPIKE distance.

## Results

### Optimal Social Interaction Is Associated With Reduced Movement Variability

**FIGURE 5:**
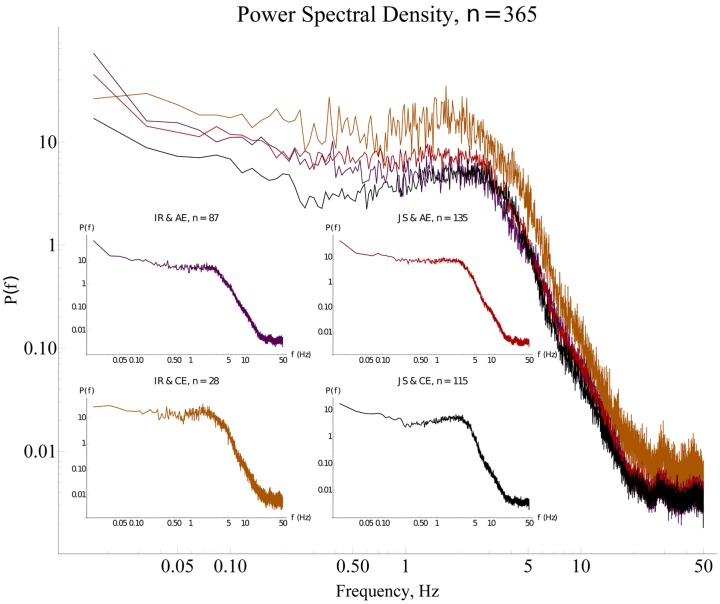
**Power spectra for different types of interaction.** The median Fourier spectra for the four modalities of social interaction have similar shapes with a broad maximum in the range 0.5–5 *Hz*, and negligible power for higher frequencies. The time-series from the purple and black spectra (convergent cases) show less variability as shown by the smaller area under the spectra, in contrast with the orange and red spectra (divergent cases) in which the surface beneath the spectra are bigger. Insets: The red spectrum has the highest power; the purple and the black spectra have the smallest power; and the orange spectrum has intermediate power. The *x* axes are trimmed at the maximal possible frequency’s value according to Nyquist’s theorem (see details in Section “Fourier Spectral Analysis,” and in “Fourier Spectral Analysis” of the Supplementary Material). IR, individual response; JS, joint success; AE, ambiguous experience; CE, clear experience.

**FIGURE 6:**
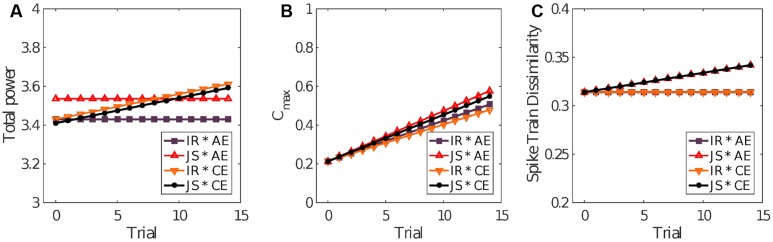
**Model predictions.** The linear mixed-effects model fits for the four scenarios determined by trial outcome according to clicks made (joint success or individual response) and the self-reported experience (clear or ambiguous). **(A)** The final models for total power, **(B)** cross-correlation, and **(C)** SPIKE-distance are shown. IR, individual response; JS, joint success; AE, ambiguous experience; CE, clear experience.

The significant coefficients in **Table 1** for the final model *Y_ij_* = *β_00_* + σ*_0i_* + *β_30_Per_ij_* +* β_40_Exp_ij_Trial_ij_ + β_60_Exp_ij_Per_ij_ + σ_ij_* of power show that power tended to be higher in JS trials by about 0.106 units, tended to increase by 0.013 units in each successive trial where *Exp* was reported, but was lower by about 0.126 units if JS and clear experience occurred in the same trial. Therefore, trials characterized by both elevated objective (joint clicking) and subjective (clear experience) sociality (convergent case of an ideal interaction) exhibited less variability in comparison to the divergent cases in which either the coordination was less successful or subjective assessment was uncertain (see **Figure 5**). The other convergent case consisting of both reduced objective and subjective sociality was also correlated with reduced variability. **Figure 6A** shows the form of the fitted model. The complete statistical outcomes of the model can be found in the Supplementary Table S1.

**Table 1:** Linear mixed-effects modeling (LMEM) for total power, cross-correlation and spikes distances.

	Total power	Cross-correlation	SPIKE-distance
**Fixed effects**
β_00_: Intercept	**3.430 (0.066)^∗∗∗^**	**0.213 (0.008)^∗∗∗^**	**0.314 (0.006)^∗∗∗^**
β_10_: Trial	0.005 (0.005)	**-0.002 (0.001)^∗^**	0.000 (0.000)
β_20_: Exp	**-**0.045 (0.069)	**-**0.011 (0.019)	0.014 (0.010)
β_30_: Per	**0.106 (0.047)^∗^**	**0.021 (0.008)^∗∗^**	0.000 (0.006)
β_40_: Exp^∗^Trial	**0.013 (0.006)^∗^**	**0.005 (0.002)^∗∗^**	**-**0.001 (0.001)
β_60_: Exp^∗^Per	**-0.126 (0.058)^∗^**	**-**0.024 (0.015)	**-0.003 (0.007)**
β_50_: Per^∗^Trial	**-**0.007 (0.006)		**0.002 (0.001)^∗^**

*^∗∗∗^p < 0.001, ^∗∗^p < 0.01, ^∗^p < 0.05. The models are specified in terms of the fitted predictor parameters and random effects. Parameter standard errors are shown in brackets. Participant (PP) acts as a grouping factor. To interpret the coefficients, consider that both clear experience and joint click success were treated as predictors of the outcome variable and coded as time-dependent binary variables. Experience (Exp) = 0 indicated in a given trial means no clear experience and Exp = 1 clear experience. Similarly, in each trial Performance (Per) = 1 if both members produced a correct response and Per = 0 otherwise; the coefficient interpretation is analogous. Trial indicates a practice effect and an interaction with Trial indicates a condition-dependent practice effect.*

### IEI Power-Law Distribution

**FIGURE 7:**
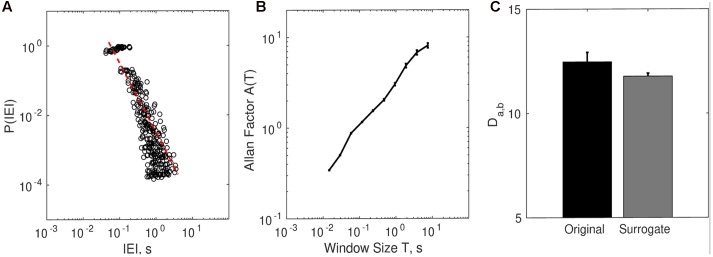
**Complexity matching in dyadic embodied social interaction. (A)** The distribution of inter-event intervals (IEI) contains the bin-averaged probability *P (IEI)* for each participant**. (B)** The relation between Allan Factor and window size for each participant averaged (SE) across all subjects and all trials. **(C)** Complexity matching for all original dyad trials (15 trials × 17 teams) and surrogate dyad trials.

The distributions of IEI were consistent with a power-law or heavy tail distribution (see **Figure 7A**). The exponent γ quantifying the *P (IEI) ∼ IEI^γ^* relation was calculated separately per each trial and then the average was obtained. The average scaling relation γ (*M* = -2.01, *SD* = 0.23) was consistent with the premises associated with complex systems (West et al., 2008). Similarly, the distribution of non-movement intervals also obeyed an inverse scaling relation with *γ* = -2. Note that this wide distribution of non-movement intervals implies that the motor behavior is not a 1/*f* process (for details on this issue, see *Distribution of Movement and Non-movement* of the Supplementary Material).

### Allan Factor Scaling and Complexity Matching in Real Embodied Dyadic Social Interaction

As **Figure 7B** suggests, on average the Allan factor of all individual times series scaled with clustering window size *T* as a power-law with a positive exponent.

The surrogate analysis showed that complexity matching among dyads (teams) was higher than expected by chance (**Figure 7C**). The average *D_a,b_^Original^* (*M* = 12.468, *SD* = 1.888) was higher than surrogate *D_a,b_^Surrogate^* (*M* = 11.779, *SD* = 0.595), *t*(16) = 1.877, *p* < 0.05.

No association between *D_a,b_* and clear experience, JS, or practice was found (results not shown) when applying the linear-mixed modeling procedure as previously described. The different types of interaction shown in **Figure 2** were not characterized by different levels of complexity matching between the two members of the dyad.

### Complexity Matching Is Not Driven by Local Coordination Patterns

#### Cross-Correlation

The surrogate analysis showed that cross-correlation between real dyads was not different from the cross-correlation of the surrogate pairs. The average for the peak positive correlation *D_a,b_^Original^* (*M* = 0.215, *SD* = 0.020) was not significantly different than the average for the surrogate pairs *D_a,b_^Surrogate^* (*M* = 0.215, *SD* = 0.009), *t*(16) = -0.010, *p* = 0.992. The same outcome was observed for the peak negative correlation *D_a,b_^Original^* (*M* = -0.170, *SD* = 0.015), when compared to the surrogate *D_a,b_^Surrogate^* (*M* = -0.167, *SD* = 0.008), *t*(16) = -0.782, *p* = 0.446.

There was no correlation between cross-correlations of movement durations and complexity matching: *r*(253) = -0.069, *p* = 0.275 for the peak positive correlation, and *r*(253) = -0.012, *p* = 0.849 for the peak negative one.

The significant coefficients in **Table 1** for the final model *Y_ij_* = *β_00_* + σ*_0i_* + *β_30_Per_ij_* +* β_40_Exp_ij_Trial_ij_ + β_60_Exp_ij_Per_ij_ + σ_ij_* of the IEI cross-correlations show that JS trials were associated with cross-correlation 0.021 units higher than in non-JS trials. Furthermore, cross-correlation tended to decrease with trials by a very small amount of 0.002 but increase by 0.004 units over trials that also resulted in clear experience report (*Exp*). **Figure 6B** shows the form of the fitted model. An expanded version of these results is provided in the Supplementary Table S2.

Motivated by the statistics showing higher peaks in *JS* trials, we repeated the surrogate analysis to compare peak cross-correlations in all *JS* trials against surrogate pairs. For the peak positive the average was *M* = 0.2231 (*SD* = 0.0526); and for the peak negative one was *M* = 0.2112 (*SD* = 0.0246). The comparison between these averages yielded a small but significant effect: *t*(130) = 2.3471, *p* = 0.0204. Note that to increase power we used all trials (*n = 131*) vs. the trial-averaged data as we did with *D_a,b_*.

#### Synchronization and SPIKE-Distance

**FIGURE 8:**
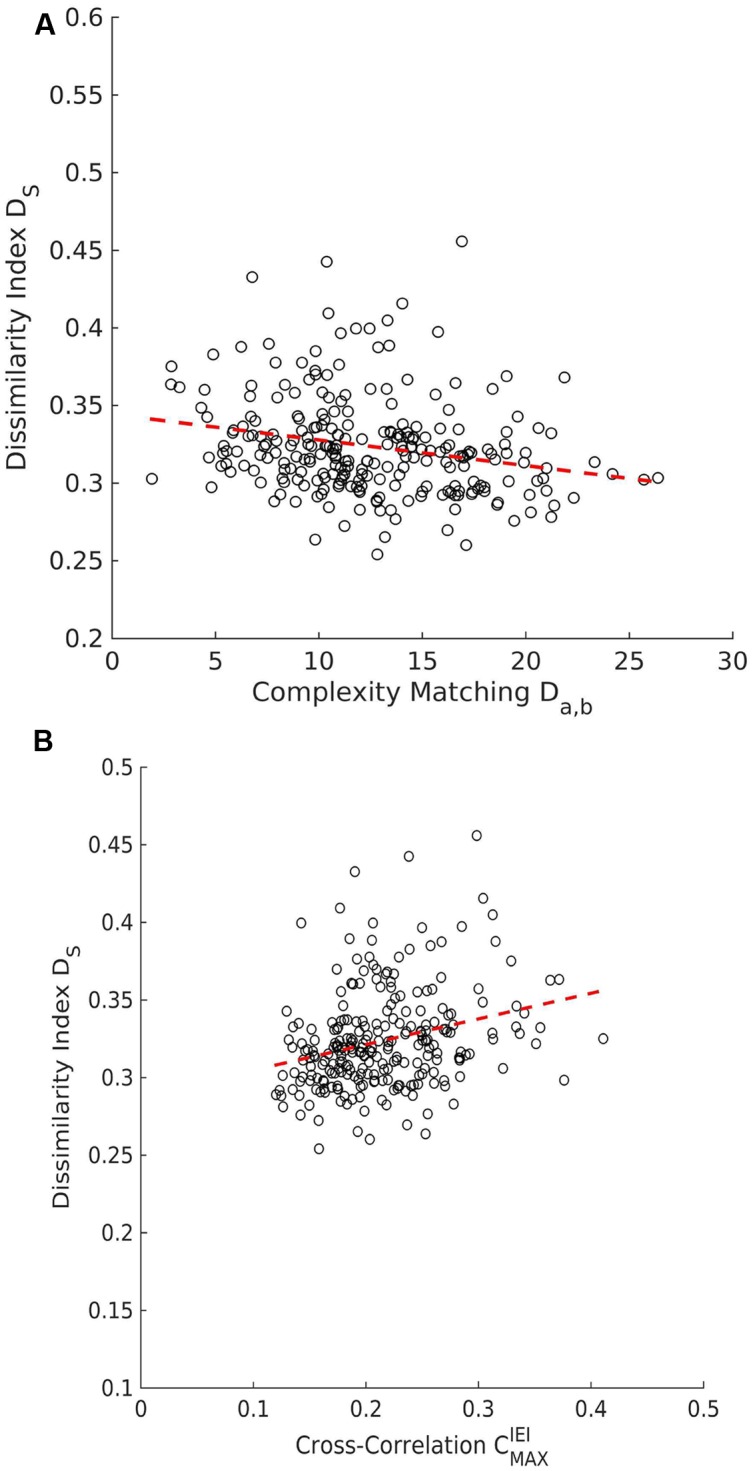
**Scatter plots for SPIKE-distance. (A)** There is a negative correlation between the dissimilarity index **D_s_** and complexity matching **(D_a,b_)_._** **(B)** The dissimilarity index **D_s_** and the maximum peak of the cross-correlation of IEI 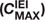 show that are positively correlated positive correlation.

A statistical test for direct event synchronization consisted of the same procedure using surrogate pairs that was also applied to the complexity matching measure. The null-hypothesis of no synchronization was not rejected, the average SPIKE-distance metric for real pairs (*M* = 0.324, *SD* = 0.022) was not different from the one for surrogate pairs (*M* = 0.323, *SD* = 0.006), *t*(16) = 0.30, *p* = 0.77. Consequently, direct synchronization between the movement start/stop events of the two members did not occur in the PCE task in the present study. A small but significant negative correlation between SPIKE-distance and complexity matching D_a,b_ was observed across all trials, *r*(253) = -0.237, *p* < 0.001, see **Figure 8A**. A small but significant positive correlation between SPIKE-distance and IEI cross-correlations was observed across all trials, *r*(253) = 0.276, *p* < 0.001, see **Figure 8B**.

The significant coefficients in **Table 1** for the final model *Y_ij_* = *β_00_* + σ*_0i_* + *β_50_Per_ij_Trial_ij_ + σ_ij_* of SPIKE-distance show that dissimilarity tended to increase but only in the JS trials, by about 0.002 units per trial. **Figure 6C** shows the form of the fitted model. The surrogate test was repeated to compare JS trials against surrogate pair trials. In this case there was a very small difference in the means of the original (*M* = 0.334, *SD* = 0.034) and surrogate pairs (*M* = 0.321, *SD* = 0.013) which, however, was statistically significant, *t*(130) = 4.31, *p* < 0.001, in a paired-samples *t*-test using all trials and participants as samples. The complete information regarding this model is included the Supplementary Table S3.

## Discussion

### Dynamical and Scaling Accounts of Social Interaction

In the present study a time-series approach to dyadic embodied social interaction was adopted as a response to the critical claim by Bedia et al. (2014), who pointed out that the previous PCE analyses were limited to a single time-scale, failing to account for the whole dynamics of dyadic social interaction. Specifically, Bedia et al. (2014) used to DFA and multifractal DFA to show that the fluctuations of such an interaction followed a power law distribution characteristic of 1/f^β^ noise (with *β≈1*), as well as that this type of interaction could be considered as a multifractal system in which multiple scales were involved, meaning that more than one scaling exponents would be needed to fully characterize the dynamics of the system (Kantelhardt, 2008).

Therefore, the objective of our research was to study the dynamics of the social interaction in the above mentioned minimal cognition environment, and particularly to look for scaling properties in the time-series obtained from the modified version of the PCE carried out by Froese et al. (2014a). In such experimental setup the players were required to perform a joint action consisting of finding and recognizing each other in a shared virtual space. A human-computer interface allowed the creation of changing sensorimotor loops so that the players could socially interact in real time and in an embodied fashion.

The series of the movements that each dyad made throughout the virtual space during the trials were analyzed with various techniques to assess different domains of the signals, namely time, frequency and scaling. These types of analyses were carried out as a complementary study to the previous single scaled behavioral analyses made by Froese et al. (2014a).

Consistent with the body of literature that acknowledges the ubiquity of multiple scales and power-law distributions in biological systems, as well as in human behavior (Ihlen and Vereijken, 2010; Kello et al., 2010; Bedia et al., 2014), we found a multi-scale pattern in the current study. Specifically, both frequency and amplitude of the time-series here assessed notably change over different time windows entailing that the dynamics of the behavioral data depend on the scale that is being taken into account. Therefore, our study supports the intuition (Bedia et al., 2014; Schmidt et al., 2014) that dyadic social interaction analysis should not be limited to short time scales alone and that a thorough evaluation (such as time-series analysis) of the phenomenon is required in order to gain a better understanding of it. In Sections “Movement Invariants in the Frequency and Time Domains Classify Social Interaction” and “PCE As a Minimal Dyadic Conversation,” the different types of analysis applied in this research are discussed.

### Movement Invariants in the Frequency and Time Domains Classify Social Interaction

The amplitude and the speed of variation over time (frequency) are essential components of every signal and to measure them is usually the first step when aiming to analyze a time-series. Accordingly, a first coarse-grained inspection of such properties was carried out. Different frequency scales of motor activity were present. Examples of these include: fast fluctuations; oscillations around a hypothetical object (which could be the other human-avatar, its shadow, or the static decoy); slow steady movement through the space; or sustained periods with absence of movement.

Afterward, we decided to study in detail the basic power spectra and the global variance of the signals. The most conspicuous finding was that different types of social interaction (in terms of objective and subjective evaluations) have distinct Fourier power spectra and thus, classifying the modalities and quantifying their differences in terms of the signals’ amount of variability were possible.

In the present context the frequency range 3–5 *Hz* in the power spectra likely corresponds to the “palpitating” rapid oscillations of one avatar around the other one. The low frequency range 0.5–3 *Hz* corresponds to slow swaying movements through the whole virtual space when one avatar is trying the find the other one. The fraction P(3 - 5Hz)/P(0.5 - 3Hz) might be a quantification of the “functional time” spent on interaction to “dead time” spent on searching.

Regarding the variability domain, the lower variance in the velocities time-series means that a slower rate of change in positions is happening. Therefore, relatively constant and low amplitude oscillations around a particular value (in this case, zero) are yielding rather stable dynamics, in which abrupt changes are absent and constant fluctuations are more likely. These properties of the time-series are consistent with the gestures observed and also previously studied in Froese et al. (2014b).

The statistical analysis shown in **Table 1** suggests that clear experience by itself is initially unrelated to the variability of movement, but over trials becomes associated with increased variability. JS by itself is also related to increased variability, but with no effect of trial-by-trial practice. Intriguingly, when JS is associated with clear experience we find a decrease in variability, which is the opposite of what we might expect from their combination. The fact clarity of experience was associated with increasing amount of activity (total variability of movement) over subsequent trials is consistent with the diachronic analysis performed by Froese et al. (2014b), which showed an increase in the frequency of reporting of clear experiences over trials (e.g., their **Figure 6**), as well as an increase in the variability of movements or at least not a simple convergence on regular turn-taking interactions.

However, we need to keep in mind that only this latter effect is indicative of a convergent situation of mutually veridical perceptual experience, whereas clear experience by itself can include false experiences, while JS by itself can include veridical interactions that were ambiguously experienced. In other words, both the divergent possibility that there is a clear experience of the other which is not veridical, and that a judgment is veridical but not based on a clear experience, are related to increased variability. Veridical perception of each other, i.e., an interaction in which both participants are clearly perceived and identified in a correct manner, stands out as a special case related to reduced variability in movement.

This makes sense because when the players do not succeed in terms of interaction, it means that their movements across the environment are not being sufficiently meaningful to each other. For instance, interactions that are less stable tend to suddenly and also more frequently break up. It is also worth mentioning that there is a limit to the reduction in variability because such minimal variability and the concomitant rigidity would indicate a non-interactive behavior as there would be no change of the individual whatsoever. This could be seen in the hypothetical case a player decides to just oscillate for the whole the trial around the static object.

Indeed, such a rigid behavior can be thought as a dysfunction in terms of social interaction, which would be expected for instance in individuals with autism spectrum disorder (ASD). Such individuals are characterized by an abnormal social approach like failure to initiate or respond to social interactions neglecting the contingencies of the environment, as well as deficits in understanding gestures, such as turn-taking. Also autistic patients present obsessive traits restricting and repeating their patterns of behavior, such as stereotyped movements or extreme distress at small changes and difficulties with transitions (American Psychiatric Association [APA], 2013). Intriguingly, perhaps the PCE could be used to characterize and even diagnose autism on the basis of time-series analyses of their embodied social interaction.

An important aspect to consider in this particular experimental set up is the fact that the presence or absence of a click is closely related to and even a direct consequence of the awareness. Indeed the recently developing field of second-person neuropsychiatry suggests that making sense of each other’s and engaging in social interaction entails a dynamical and non-detached process (Schilbach, 2016). So whereas the clicks in the PCE are single scale point events, perception of the environment can be regarded rather as a flow of integrated and continuously active engagement that will or will not eventually create an impression of the situation. It is plausible to assume that the trajectory of this dynamical process will yield an impression only if two conditions are met:

(1) Sufficient attention is paid to the sensorimotor loop in which the players are being entrained; and
(2) Co-regulation is reached, so the collective modulation of the entrained sensorimotor loop is constantly maintained.

In this sense, the reduced variability can also be interpreted as focalized and stable attention which would explain why it is more related to accurate awareness (convergent situations) rather than with clicking accuracy. Moreover, our results suggest that such dynamics allow the players to detect subtleties in the environment, namely the contingencies provided by the different objects encountered.

### PCE As a Minimal Dyadic Conversation

It is important to mention that the study by Bedia et al. (2014) cannot be compared directly with the present one because of some differences in the experimental setup. The most relevant are: the lack of both a joint action task and a tournament based competition; and the fact that participants could only encounter one type of object in the virtual space.

Even though such discrepancy, we searched for long-range correlations and fractal features in the fluctuations of the velocities time-series. To do so we attempted to apply Detrended Fluctuation Analysis (results not shown), a method that has been used for studying continuous movement fluctuations such as, for instance, posture control (e.g., Duarte and Zatsiorsky, 2000). However, our data set suggest that such measures were not meaningful in the current PCE paradigm due to the composition of a fluctuating signal and constant signal. Instead we consider that a more appropriate frame of reference is to treat PCE as a form of conversation and point processes that require different methods such as the clustering statistics of discrete actions or discrete events.

Worth to mention is that in contrast to Bedia et al. (2014) considering the relative velocities as a proxy of collective behavior was disregarded in the current research. This decision is based upon the finding that such series did not provide any additional information about the dynamics, and even caused the loss of patterns related to the social interaction itself (results not shown). For instance, turn-taking gestures disappeared after compacting the two individuals’ series into a “collective” one. Importantly, such patterns can be considered as a form of synchrony between players. Therefore, we avoided losing such information as our main purposes in this study were: on the one hand, to characterize situations in which people make sense of each other; and on the other hand, to potentially generate a behavioral marker of successful social interaction integrating both objective and subjective perspectives.

In the present study we consider the possibility that the PCE converges not only methodologically but also theoretically with the multi-scale coordination that is affiliative dialog. This possibility is consistent with the claim that dialog is a joint action at different levels, including non-linguistic or embodied aspects (Garrod and Pickering, 2009). Besides, it is clear that in both the PCE and dyadic conversations two participants are collaboratively solving a problem. Collaborative interaction implies that the partners need to coordinate each other’s activity. This activity is embedded inside a single communication dimension (acoustic pressure wave in the conversation and lateral excursion of the pointer in the perceptual crossing setup). Furthermore, the paradigms for studying complexity-matching in conversational styles (Abney et al., 2014; Coey et al., 2016) and the present perceptual crossing study contain important similarities in terms of the abstract description of their task spaces.

Indeed, by adopting such a perspective we found a power-law distribution of the IEI when considering the series in binary form (point processes). The Allan factor coefficients of such series also showed a power-law distribution, compatible indeed with multi-scale dynamics in the behavioral activity of the players during the PCE social interaction. In addition, the comparison between different Allan factor coefficients within dyads showed the presence of the so-called complexity matching. Additionally, these distribution similarity indices were compared to the respective ones obtained from surrogate dyads and revealed a statistical significant difference, meaning that our finding is different from the expected by chance, and therefore we regard this measure of synchrony between dyads interaction as a marker of real-time social interaction under this experimental setup. It is interesting though, that complexity matching was not significantly different for trials that gave rise to a clear experience of the other and/or jointly successful clicks. It therefore seems to measure a more general alignment of activity that is independent of subjective impression and objective performance of mutual recognition. This notion is compatible with the results presented by Coey et al. (2016), that posit complexity matching as a more general rather than specific behavioral marker.

However, as it has been mentioned previously, it might be the case that complexity matching arises from local coordination patterns, rather than from higher-order dynamics. Accordingly, the SPIKE-distance and cross-correlation methods were crucial in order to confirm and strengthen our findings. The spikes distance measures direct synchronization among events on an inverted scale (zero is maximum and one is no synchronization). The negative correlation between spike distances and the complexity matching metric D_a,b_ in addition to the fact that complexity matching passed the surrogate test but the direct synchronization did not, suggests that multi-scale generalized interaction processes increases the chances for local coordination to occur but local coordination is not what drives the interaction among partners in the PCE task. Thus, complexity matching is picking up something distinct that cannot be reduced to the standard measures of local coordination.

Moreover, it is not surprising that the SPIKE-distance and the IEI cross-correlations were found to be correlated since both are sensitive to direct local coordination. The positive sign of the correlation can be explained by the fact that cross-correlation can detect alignment with a lag whereas the SPIKE-distance method is time-localized. Even if JS implies the emergence of local coordination this coordination was still likely to be characterized by a delay. The cross-correlation measure can shift the series to align them whereas the measure of SPIKE-distance cannot. It follows that in joint successful trials partners managed to engage in behavior that was momentarily dissimilar but matching with a delay, consistent with turn-taking where one of the participant’s waits and “listens” for the other to move and the two switch roles.

Importantly, these finding are consistent with the claim that aspects of bodily interactive alignment during dialog can be incidental and that individuals engaged in the interaction are typically unaware of this alignment processes (Garrod and Pickering, 2009). In particular, Pickering and Garrod (2004) proposed that the most important way in which alignment occurs is via a process of automatic, i.e., non-conscious and effortless, imitation at different levels. Perhaps this explains our finding of scale-free complexity matching across all trials. It is also consistent with Froese and Di Paolo’s (2011) hypothesis that the user experience in a PCE only takes on a social quality when interaction is co-regulated in such a way that an action’s conditions of success and failure are distributed across the interactors, a situation which may have to be measured in terms of local coordination methods.

The PCE is a novel and unique task. The multi-scale coordination behavior that makes complexity matching possible needs further investigation. Observation of participants’ behavior suggests that they engaged in several kinds of sensorimotor coordination patterns characterized by different time-scales. Examples of these include fast fluctuations, oscillations around a hypothetical avatar (which could be real, its shadow, or the static decoy), slow steady movement through the space, or periods of sustained non-movement.

Complexity matching accounts for the fact that partners tended to match the relative distributions or frequencies of these coordination patterns within a trial. These patterns were not aligned as implied by the lack of local coordination. Cross-correlations and spike-coincidence require that the separate movement patterns are matched not only in frequency but also in order between partners. In fact, the emergence of local coordination early in the experiment would be unexpected because participants were not continuously coupled but only received feedback of each other’s activity sparingly and in categorical manner. There was some evidence, however, that local coordination tended to appear in later trials when those were jointly successful. This suggests that participants were beginning to discover a form of simple local coordination which allowed them to guess each other’s presence. Thus, exploring of the task space and searching for the partner in a very limited and uncertain medium are complex multi-scale processes but the successful bounding between partners consists of the emergence of a local coordination pattern akin to joint synchrony. The performance is complex but the finale is simple.

### Future Work and Potential Clinical Implications

This is among the first studies to find evidence for a longer time scale of coordination emerging in the PCE as a paradigmatic instance of real-time embodied social interaction, following the pioneering study by Bedia et al. (2014). The matched multi-scale clustering we found in our analysis indicates a behavioral dynamics extending beyond the immediate response to isolated perceptual crossings or an individual short-scale strategy. In the context of conversation, complexity matching is interpreted as coordinated alignment at multiple levels of verbal communication such as individual acoustic events, words, sentences, turn taking, etc. (Abney et al., 2014; Fusaroli et al., 2014), which is likely to extend to non-verbal aspects of dialog as well (Garrod and Pickering, 2009).

The significant interaction that we found between clear social experience and jointly successful clicking suggested such relation could not have been explained by either clear social experience or JS separately. Such results support ecological and phenomenological accounts of genuinely inter-subjective experience because it allows for the possibility that the actual coupling between the participants at least partially co-constitutes their mutually shared awareness. Indeed, such a relational interpretation would also help to make sense of the otherwise surprising fact that reduced variability of movements not only picks out situations of correctly shared clear social awareness, but is additionally characteristic of trials in which IRs are paired with ambiguous social awareness. And if the presence of an inter-subjective relation can be constitutive of a certain mode of cognition, perhaps the absence of that relation could be similarly constitutive of perceiving a lack of another person.

Remarkably, time-series analysis in the frequency and variability domains quantified such a dyadic relation, suggesting that this type of analysis could enlarge the scope for studying such a phenomenon and it could also be of help for considering relevant properties of embodied interaction that simpler and less thorough assessments would neglect. Clearly, there is much to be done in order to get a deeper understanding of social interaction. To this end various additional methods might be of the utmost relevance, such as the cross-wavelet transform for measuring time-frequency couplings (Issartel et al., 2015), phase space reconstruction and cross recurrence quantification analysis for capturing synchronization as well as synergetic and non-linear properties (Kantz and Schreiber, 2004; Fusaroli et al., 2014).

Crucially, the PCE as an embodied social interaction paradigm enables to study from a minimally cognition perspective both motor and sensory aspects, although here we limited our investigation to motor aspects alone. We expect that key characteristics of the sensorimotor time-series would be different when produced by participants with social impairments. Therefore, time-series analysis in this context could be extended into the realm of social interaction disorders to search for potential behavioral markers that would help clinicians for a better assessment of patients suffering from a social interaction disorder. Particularly, time-series analysis of the movements has been proved useful for detecting subtle traits like hindered synchronization and patients with ASD when compared to healthy controls (Fitzpatrick et al., 2016).

Moreover, due to the contingencies that are located in the PCE virtual space, it is possible to also investigate the role that the environment is playing in individuals with embodied social interaction impairments like Möbius syndrome (Michael et al., 2015) or ASD, for in the latter case there is thought to be a dissociation between what is taking place in the environment, and what is being perceived by the patients. So even though the objects are the same for both players, the affordances that would be enacted would be hypothetically different based upon the sensorimotor loop sustained throughout the interaction itself. Such an endeavor could bring some light into the flourishing fields of social neuroscience and second-person psychiatry, which have been intensively studying real-time interaction between actively engaged subjects, and also have suggested that social phenomena should be understood and investigated in terms of their dynamics across different timescales (Schilbach et al., 2013).

## Author Contributions

LZ-F and DD wrote the initial manuscript. LZ-F, RF, and DD analyzed the data. DD performed the statistical analyses. All authors developed the hypothesis, discussed the results, and worked on the manuscript.

## Funding

We acknowledge funding from PAPIIT-DGAPA-UNAM (grants IN113013, IN106215 and IV100116), CONACYT (grants 221341 and 167441) and the Newton Advanced Fellowship from the British Academy of Medical Sciences.

## Conflict of Interest Statement

The authors declare that the research was conducted in the absence of any commercial or financial relationships that could be construed as a potential conflict of interest.
