# Supplementary material for: Time-Series Analysis of Embodied Interaction: Movement Variability and Complexity Matching As Dyadic Properties
Source: Front Psychol. 2016 Dec 12;7:1940. doi: 10.3389/fpsyg.2016.01940 (PMC5149553; doi:10.3389/fpsyg.2016.01940)
Supplement: Supplementary file 1 [file Data_Sheet_1.docx]

Supplementary Material

Time series analysis of embodied interaction: Movement variability and complexity matching as dyadic properties

Leonardo Zapata-Fonseca*, Dobromir Dotov, Ruben Fossion, Tom Froese

*** Correspondence:** Leonardo Zapata-Fonseca: zapatafonseca.leonardo@gmail.com

# Fourier spectral analysis

As a common practice, when assessing a time-series, the first step is to compute the Fourier power spectra. Accordingly, in the current study we firstly applied the Fourier transform to the individuals’ time-series of instantaneous velocities.

The Fourier transform is a linear transformation that decomposes a discrete time-series *x(n)*=*x_1_*, *x_2_*, … *x_N_* of *N* successive observations as the sum of periodic basis functions $e^{i\omega_{k}n}=\cos\left( \omega_{k}n \right)+i\sin\left( \omega_{k}n \right)$ (Butz, 2006),

$$x\left( n \right)=\sum_{k=0}^{N-1} X_{k}e^{i\omega_{k}n}$$

where *X_k_* are complex Fourier coefficients that serves as weights for the periodic functions,

$$X_{k}=\frac{1}{N}\sum_{n=1}^{N} x(n)e^{-i\omega_{k}n}$$

where the frequency ω*_k_*=±2π*k*/*N* indicates *k* complete cycles over the whole duration of *N* datapoints and where frequencies can be positive and negative. The Nyquist theorem establishes that the maximal frequency is ω*_k_*=±2π*(N*/2*)*, i.e., *N*/2 complete cycles over the whole duration of the time series with length *N*, or 1 cycle every 2 data points.

Given the nature of such cyclical and trigonometric functions, the signal can be represented as a circular path inside a Euclidean space. Therefore, the features of this circular object are crucial for understanding the time-series in its frequency domain perspective.

Firstly, the size of the circle (i.e., radius’ size) accounts for the amplitude of the time-series. Secondly, the speed in which the circular path repeats itself becomes the frequency of the signal (the faster the cycle, the higher the frequency). Thirdly, the starting point of the cycle is known as the phase of the signal; in terms of Euclidean geometry it corresponds to the angle (with respect to the x-axis) at which the outlining/drawing of the circular path begins.

Once the transformation is applied, it yields the so-called Fourier power spectrum,

$$P\left( k \right)=\left\{ \left| X_{k} \right|^{2}, k=0,\ldots,N-1 \right\}$$

which is then the collection of the power |*X_k_*|^2^ contained in each of the periodic basis functions and where the powers are ordered according to frequency *ω_k_*. The power |*X_k_*|^2^ can be interpreted as a partial variance, and Parseval’s theorem establishes that cumulative sum over all partial variances equals the total variance (Var) of the original time series,

Notice that Fourier transform adds complex numbers; however, the usual programming languages cannot handle complex numbers directly and therefore another transformation is required for being able to actually compute the decomposition.

The main advantage of Fourier spectral analysis is that the single value of the total variance Var of the time series is decomposed in contributions at different frequencies, which allows distinguishing between time series with equal Var but dominant contributions from different frequency ranges. Moreover, the power spectrum also can be used for assessing power-law distributions which would indicate that low frequencies or slow waves contribute more to the series than high frequencies or rapid waves (Muñoz-Diosdado et al., 2005). Such a behavior is formalized by the relation ${P\left( k \right)\propto1}/{k^{\beta}}$ , where the scaling exponent ***β*** is the slope of the fitted line in the double logarithmic plot of the power spectra.

# Distribution of movement and non-movement

Singularity is a mathematical term with fundamental importance in many fields of science. It designates a failure of the analytical expression of a given process. In time series, a singularity is a point of discontinuity where the derivative(s) is not defined because the curve at that point is not-smooth, i.e. the process cannot be described locally with ordinary differential equations, polynomial fitting, or a Fourier series. Self-affine series, more commonly referred to as fractals in time, are time series each point of which is a singularity (Mandelbrot, 1983).

DFA and other methods in its category are designed to quantify the scaling properties of singular series with the basic assumption that the series is singular everywhere (Peng et al., 1994). DFA does not have a preliminary step to check this assumption. Other methods such as WTMM make it easier to check this assumption because they can be used to visualize the singularities directly along with the original time series (for a telling example see Ivanov et al., 1999; also the guide intended to describe the linkage between singularity and scaling, <https://www.physionet.org/tutorials/multifractal/>).

The silent periods in the participant’s movement in the PCE task are perfectly smooth and in this way they fail to satisfy the assumption of singularity. Note the wide distribution of silences in Supplementary Figure 1. Silences up to ten seconds were even possible and the cumulative duration of non-movement periods lasting 100 milliseconds or more was 42% of the total trial, averaged across all participants and trials. In this context, removing the silent periods recorded in the PCE task and concatenating the active parts is not an option either. This operation would create a great number of spurious discontinuities at the start of each concatenated block and greatly bias the procedure.

# SPIKES-distance

This method is normally used in neuroscience in order to assess local coordination. To summarize it briefly, it computes an overall spike distance profile function *S* for each sample of the binary input signals based on the distances among the spikes. It first takes the distances between each spike, called a corner spike, in the one series and the closest previous and following spikes in the other series, $\Delta{t_{P}}^{\left( 1 \right)}={min}_{i}\left( \left| {t_{P}}^{\left( 1 \right)}-{t_{i}}^{\left( 2 \right)} \right| \right)$ and $\Delta{t_{F}}^{(1)}={min}_{i}(|{t_{F}}^{(1)}-{t_{i}}^{(2)}|)$. The inter-train spike distances are weighted locally at each sample time *t* by distances ${x_{P}}^{\left( 1 \right)}\left( t \right)=t-{t_{P}}^{\left( 1 \right)}(t)$ and ${x_{F}}^{\left( 1 \right)}\left( t \right)={t_{F}}^{\left( 1 \right)}\left( t \right)-t$ to the nearest preceding and following spike of the same train. The following equation gives the instantaneous spike distance of the one train.

$$S_{1}=\frac{\frac{\Delta{t_{P}}^{\left( 1 \right)}}{{x_{P}}^{\left( 1 \right)}}+\frac{\Delta{t_{F}}^{\left( 1 \right)}}{{x_{F}}^{\left( 1 \right)}}}{\frac{1}{{x_{P}}^{\left( 1 \right)}}+\frac{1}{{x_{F}}^{\left( 1 \right)}}}$$

All operations are repeated symmetrically for the other series,$\Delta{t_{P}}^{\left( 2 \right)}={min}_{i}\left( \left| {t_{P}}^{\left( 2 \right)}-{t_{i}}^{\left( 1 \right)} \right| \right)$, $\Delta{t_{F}}^{(2)}={min}_{i}(|{t_{F}}^{(2)}-{t_{i}}^{(1)}|)$, ${x_{P}}^{\left( 2 \right)}\left( t \right)=t-{t_{P}}^{\left( 2 \right)}(t)$, ${x_{F}}^{\left( 2 \right)}\left( t \right)={t_{F}}^{\left( 2 \right)}\left( t \right)-t$, and $S_{2}=\frac{\frac{\Delta{t_{P}}^{\left( 2 \right)}}{{x_{P}}^{\left( 2 \right)}}+\frac{\Delta{t_{F}}^{\left( 2 \right)}}{{x_{F}}^{\left( 2 \right)}}}{\frac{1}{{x_{P}}^{\left( 2 \right)}}+\frac{1}{{x_{F}}^{\left( 2 \right)}}}$.

The profile function is given by

$$S(t)=\frac{S_{1}(t){x_{ISI}}^{\left( 2 \right)}+S_{2}(t){x_{ISI}}^{\left( 1 \right)}}{2\left\langle{x_{ISI}}^{\left( n \right)} \right\rangle_{n}^{2}}$$

where the intra-train inter-spike-intervals for train *n* are ${x_{ISI}}^{\left( n \right)}\left( t \right)={x_{P}}^{\left( n \right)}\left( t \right)+{x_{F}}^{\left( n \right)}\left( t \right)$. A scalar metric of overall synchronization is the average over time $D_{S}=\frac{1}{T}\int_{t=0}^{T} S\left( t \right)dt$. $D_{S}$ is a dissimilarity measure and ranges from 0 when all spikes are perfectly aligned to 1 when spikes in the one train are randomly occurring with respect to spikes in the other train. Supplementary Figure 2 illustrates a sample trial from the PCE study where the spike distance profile *S* is shown along with the events of movement start and stop for each member of the pair.

# Linear mixed-effects modeling

A minimal model consisting of a constant intercept, i.e. a grand mean, was expanded by including performance according to types of clicks, (*Per*), subjective experience (*Exp*), trial number (*Trial*), and the interaction between *Per* and *Exp*, in that order, targeting the same model template for consistency and easier interpretation,

*Y_ij_* = *β_00_* + σ*_0i_* + *β_10_Trial_ij_* + *β_20_Exp_ij_* + *β_30_Per_ij_* + *β_40_Exp_ij_Trial_ij_ + β_50_Per_ij_Trial_ij_ +β_60_Exp_ij_Per_ij_ +σ_ij ._*

Here *Y* is the respective outcome variable, *i* stands for participant number, *j* for trial number, *σ_0i_* is the variability in individual baseline, and *σ_ij_* is the residual of the model. The fitted coefficients are represented by *β*’s and the predictors by self-explanatory labels. Only the significant predictors were retained in the final models. The sequence of models for total power is specified in Supplementary Table 1; for IEI cross-correlations in Supplementary Table 2; and for SPIKE-distance in Supplementary Table 3. The analysis answers the question whether subjective experience, performance and their interaction are associated with changes in the respective outcome variable and whether there was a practice effect.

The final model for movement power is expressed by the equation

*Y_ij_* = *β_00_* + σ*_0i_* + *β_10_Trial_ij_* + *β_30_Per_ij_* + *β_40_Exp_ij_Trial_ij_ +σ_ij ._*

The final model for cross-correlation of IEI is expressed by the equation

*Y_ij_* = *β_00_* + σ*_0i_* + *β_30_Per_ij_* + *β_40_Exp_ij_Trial_ij_ + β_60_Exp_ij_Per_ij_ +σ_ij ._*

The final model for SPIKE-distance is expressed by the equation

*Y_ij_* = *β_00_* + σ*_0i_* + *β_50_Per_ij_Trial_ij_ + σ_ij ._*

# Supplementary Figures

## Supplementary Figure 1. Distribution of silences


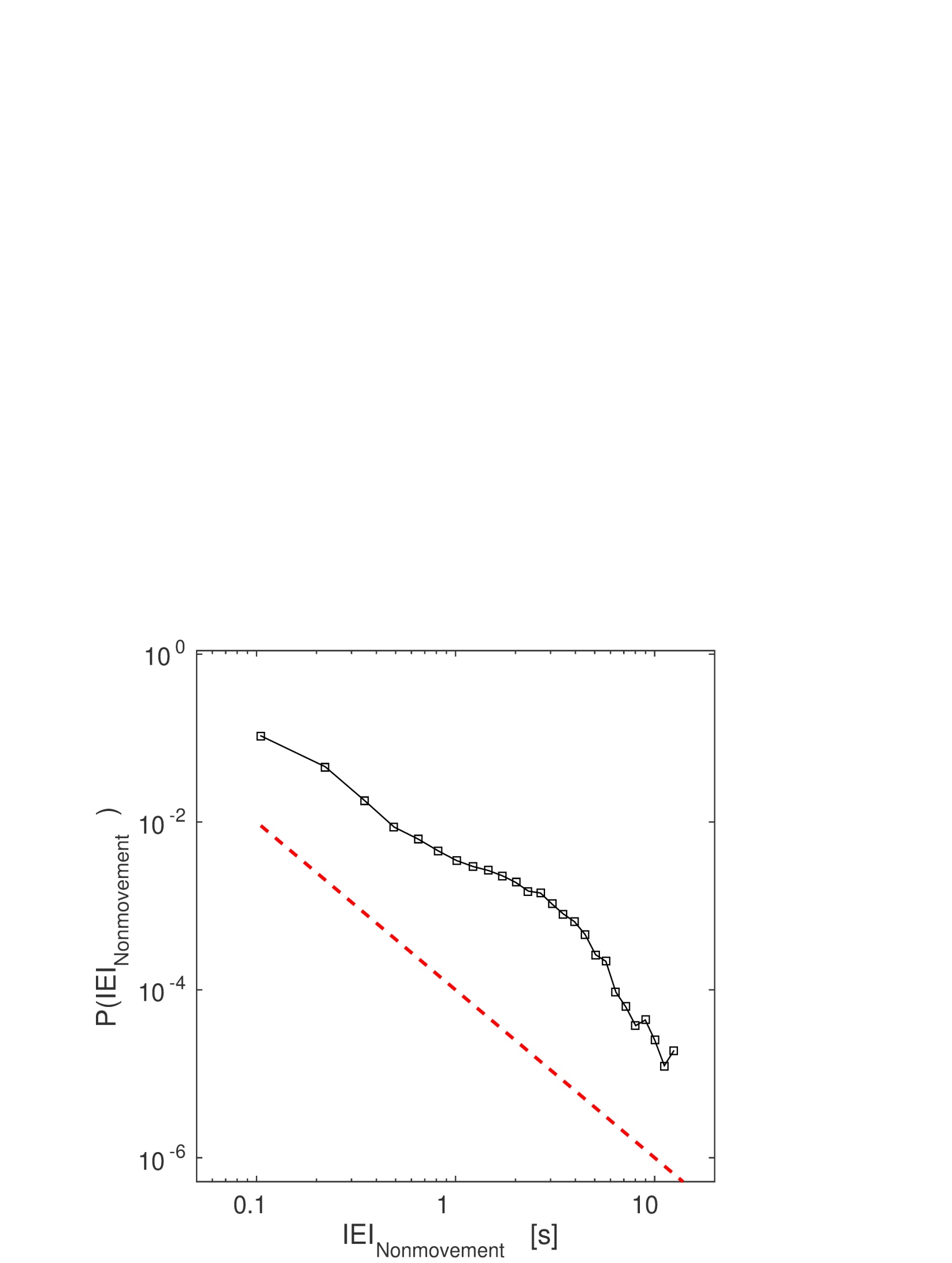


**Supplementary Figure 1.Distribution of silences.** The distribution of “silent” non-movement intervals between movement offset and movement onset is shown in terms of the histogram of all intervals pooled across trials and participants. The distribution closely follows the theoretical inverse scaling$P(IEI)={IEI}^{\gamma}$, $\gamma=-2$, shown in red that was also observed for the movement inter-event-intervals.

## Supplementary Figure 2. Representative SPIKE-distance profile


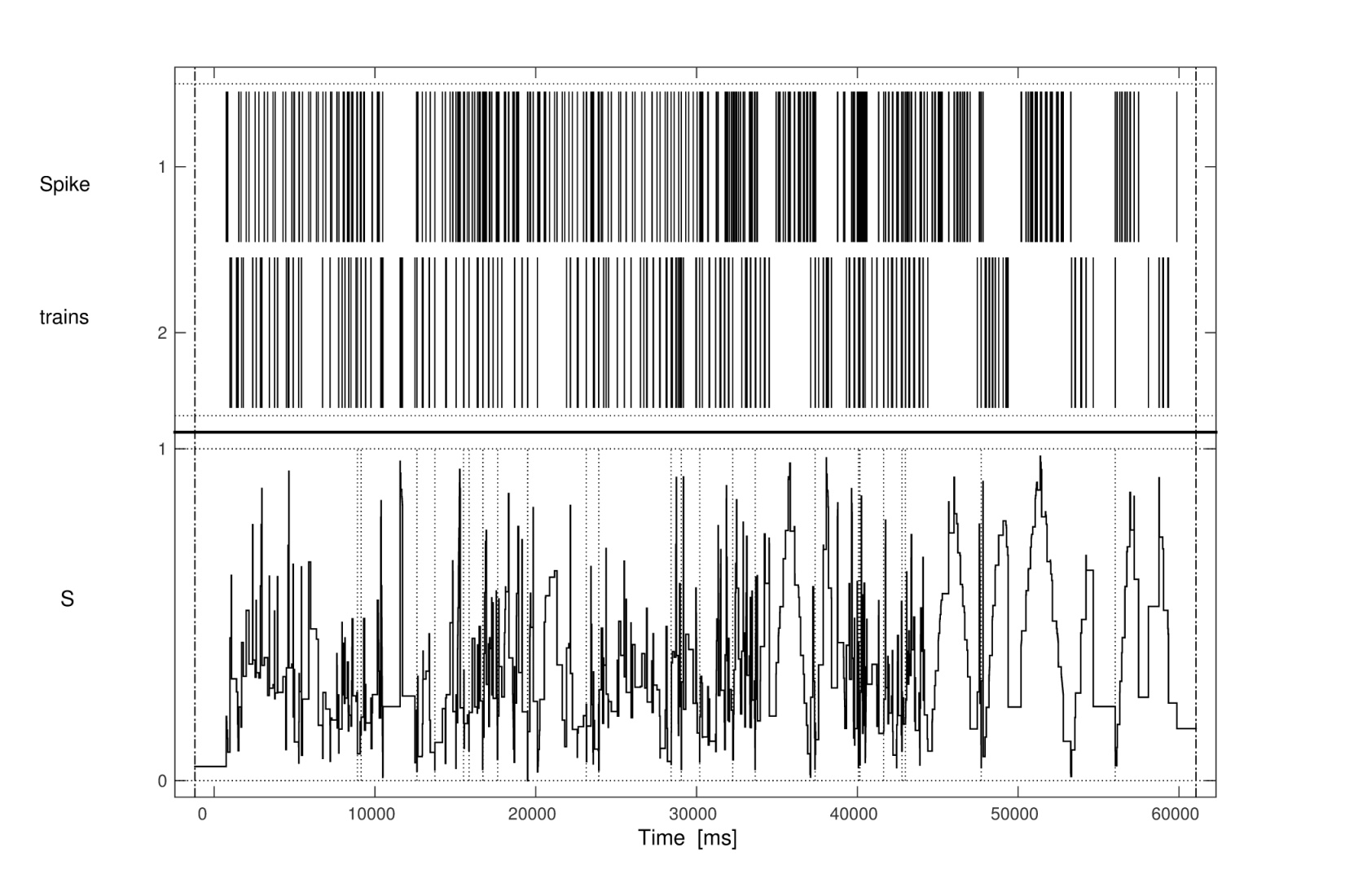


**Supplementary Figure 2. Representative SPIKE-distance profile.** The movements’ onsets and endings(events) from a sample trial are shown along with the spike distance profile *S*(*t*).

# Supplementary Tables

## Supplementary Table 1. Linear mixed-effects models (LMEM) for total power. The models are specified in terms of the fitted predictor parameters and random effects. The final model for movement power is Model 5. Parameter standard errors are shown in brackets. Participant (PP) acts as a grouping factor. To interpret the coefficients, consider that both clear experience and joint click success were treated as predictors of *the* outcome variable and coded as time-dependent binary variables*. Exp* = 0 indicated in a given trial means no clear experience and *Exp* = 1 clear experience. The predictor parameter then indicates how much increa*s_e_* in movement velocity power is associated with cle*a_r_* experience (*β_20_**1) with respec*t t*o non-clear experience trials (*β_20_**0). Similarly, in *eac*h trial *Per* = 1 if both members produced a correct response a*nd Per* = 0 otherwise; the coefficient interpretation is an*alogo*us. *Trial* indicates a practice effect and an interaction with *Trial* indicates a condition-dependent practice effect.

|  | **Model 1** | **Model 2** | **Model 3** | **Model 4** | **Model 5** |
| --- | --- | --- | --- | --- | --- |
| *Fixed Effects* |  |  |  |  |  |
| *β_00_: Intercept* | **3.460 (0.061)***** | **3.467 (0.061)***** | **3.475 (0.064)***** | **3.440 (0.067)***** | **3.430 (0.066)***** |
| *β_10_: Trial* | 0.005 (0.003) | **0.005 (0.003)*** | 0.002 (0.003) | 0.007 (0.004) | 0.005 (0.005) |
| *β_20_: Exp* |  | -0.034 (0.028) | **-0.123 (0.054)*** | **-0.138 (0.055)*** | -0.045 (0.069) |
| *β_30_: Per* |  |  | 0.023 (0.026) | 0.089 (0.046) | **0.106 (0.047)*** |
| *β_40_: Exp*Trial* |  |  | 0.011 (0.006) | **0.013 (0.006)*** | **0.013 (0.006)*** |
| *β_50_: Per*Trial* |  |  |  | -0.010 (0.006) | -0.007 (0.006) |
| *β_60_: Exp*Per* |  |  |  |  | **-0.126 (0.058)*** |
| *Variance Components* |  |  |  |  |  |
| *σ_0i_: Level-2 : PP intercept* | 0.107 | 0.107 | 0.108 | 0.107 | 0.104 |
| *σ_ij_: Level-1 : Residual* | 0.043 | 0.043 | 0.043 | 0.042 | 0.042 |
| *Goodness-of-fit* |  |  |  |  |  |
| *AIC* | 7.812 | 8.321 | 8.434 | 7.431 | 4.691 |
| *BIC* | 23.412 | 27.821 | 35.733 | 38.630 | 39.790 |
| *Log Likelihood* | 0.094 | 0.839 | 2.783 | 4.285 | 6.655 |
| *^†^ χ^2^* |  | 1.49 | 3.89 | 3.00 | 4.74 |
| *^†^ df* |  | 1 | 2 | 1 | 1 |
| *^†^ p* |  | 0.22 | 0.14 | 0.08 | 0.03 |
| *For parameters, **p < 0.001, **p < 0.01, *p < 0.05. Observations in each model = 365. Groups (PP) = 34. ^†^The goodness-of-fit test is with respect to the model standing previous in the series of models for the given outcome variable, i.e. Model 2 with respect to Model 1* | | | | | |

## Supplementary Table 2. LMEM for cross-correlation of IEI. The models are specified in terms of the fitted predictor parameters and random effects. The final model for cross-correlation of IEI is Model 6 *_._*Parameter standard errors are shown in brackets. Participant (PP) acts as a grouping factor. To interpret the coefficients, consider that both clear experience and joint click success were treated as predictors of the outcome variable and coded as time-dependent binary variables. *Exp* = 0 indicated in a given trial means no clear experience and *Exp* = 1 clear experience. Similarly, in each trial *Per* = 1 if both members produced a correct response and Per = 0 otherwise; the coefficient interpretation is analogous. *Trial* indicates a practice effect and an interaction with *Trial* indicates a condition-dependent practice effect The models were fit on 255 observations (trials) and 17 grouping levels for the random intercept corresponding to the 17 dyads

|  | **Model 1** | **Model 2** | **Model 3** | **Model 4** | **Model 5** | **Model 6** | **Model 7** |  |
| --- | --- | --- | --- | --- | --- | --- | --- | --- |
| *Fixed Effects* |  |  |  |  |  |  |  |  |
| *β_00_: Intercept* | **0.215 (0.005)***** | **0.207 (0.006)***** | **0.205 (0.006)***** | **0.202 (0.006)***** | **0.206 (0.007)***** | **0.213 (0.008)***** | **0.210 (0.009)***** |  |
| *β_30_: Per* |  | **0.016 (0.007)*** | 0.013 (0.007) | **0.019 (0.008)*** | **0.020 (0.008)*** | **0.021 (0.008)**** | **0.028 (0.013)*** |  |
| *β_20_: Exp* |  |  | 0.009 (0.008) | 0.025 (0.013) | **0.027 (0.013)*** | -0.011 (0.019) | -0.015 (0.020) |  |
| *β_60_: Exp*Per* |  |  |  | -0.023 (0.016) | -0.024 (0.016) | -0.024 (0.015) | -0.022 (0.016) |  |
| *β_10_: Trial* |  |  |  |  | -0.001 (0.001) | **-0.002 (0.001)*** | -0.001 (0.001) |  |
| *β_40_: Exp*Trial* |  |  |  |  |  | **0.005 (0.002)**** | **0.005 (0.002)**** |  |
| *β_50_: Per*Trial* |  |  |  |  |  |  | -0.001 (0.002) |  |
| *Variance Components* |  |  |  |  |  |  |  |  |
| *σ_0i_: Level-2 : PP intercept* | 0.000 | 0.000 | 0.000 | 0.000 | 0.000 | 0.000 | 0.000 |  |
| *σ_ij_: Level-1 : Residual* | 0.003 | 0.003 | 0.003 | 0.003 | 0.003 | 0.002 | 0.002 |  |
| *Goodness-of-fit* |  |  |  |  |  |  |  |  |
| *AIC* | -770.666 | -774.378 | -773.822 | -774.080 | -772.768 | -777.617 | -776.103 |  |
| *BIC* | -760.042 | -760.212 | -756.115 | -752.833 | -747.979 | -749.287 | -744.232 |  |
| *Log Likelihood* | 388.333 | 391.189 | 391.911 | 393.040 | 393.384 | 396.809 | 397.051 |  |
| *^†^ χ^2^* |  | 5.7120 | 1.4440 | 2.2586 | 0.6877 | 6.8495 | 0.4856 |  |
| *^†^ df* |  | 1 | 1 | 1 | 1 | 1 | 1 |  |
| *^†^ p* |  | 0.016 | 0.229 | 0.132 | 0.406 | 0.008 | 0.485 |  |
| *For parameters, **p < 0.001, **p < 0.01, *p < 0.05. Observations in each model = 255. Groups (PP) = 17. ^†^The goodness-of-fit test is with respect to the model standing previous in the series of models for the given outcome variable, i.e. Model 2 with respect to Model 1* | | | | | | | | |

## Supplementary Table 3. LMEM for spikes distances. The models are specified in terms of the fitted predictor parameters and random effects. The final model for SPIKE-distance is Model 7*_._* Parameter standard errors are shown in brackets. Participant (PP) acts as a grouping factor. To interpret the coefficients, consider that both clear experience and joint click success were treated as predictors of the outcome variable and coded as time-dependent binary variables. *Exp* = 0 indicated in a given trial means no clear experience and *Exp*= 1 clear experience. Similarly, in each trial *Per* = 1 if both members produced a correct response and *Per* = 0 otherwise; the coefficient interpretation is analogous. *Trial* indicates a practice effect and an interaction with *Trial* indicates a condition-dependent practice effect.

|  | **Model 1** | **Model 2** | **Model 3** | **Model 4** | **Model 5** | **Model 6** | **Model 7** |  |
| --- | --- | --- | --- | --- | --- | --- | --- | --- |
| *Fixed Effects* |  |  |  |  |  |  |  |  |
| *β_00_: Intercept* | **0.324 (0.005)***** | **0.316 (0.005)***** | **0.314 (0.005)***** | **0.314 (0.005)***** | **0.310 (0.006)***** | **0.310 (0.006)***** | **0.314 (0.006)***** |  |
| *β_30_: Per* |  | **0.015 (0.003)***** | **0.013 (0.003)***** | **0.013 (0.004)**** | **0.012 (0.004)**** | **0.012 (0.004)**** | 0.000 (0.006) |  |
| *β_20_: Exp* |  |  | **0.009 (0.004)*** | 0.009 (0.006) | 0.006 (0.006) | 0.007 (0.009) | 0.014 (0.010) |  |
| *β_60_: Exp*Per* |  |  |  | 0.000 (0.007) | 0.001 (0.007) | 0.001 (0.007) | -0.003 (0.007) |  |
| *β_10_: Trial* |  |  |  |  | **0.001 (0.000)*** | 0.001 (0.000) | 0.000 (0.000) |  |
| *β_40_: Exp*Trial* |  |  |  |  |  | -0.000 (0.001) | -0.001 (0.001) |  |
| *β_50_: Per*Trial* |  |  |  |  |  |  | **0.002 (0.001)*** |  |
| *Variance Components* |  |  |  |  |  |  |  |  |
| *σ_0i_: Level-2 : PP intercept* | 0.000 | 0.000 | 0.000 | 0.000 | 0.000 | 0.000 | 0.000 |  |
| *σ_ij_: Level-1 : Residual* | 0.001 | 0.001 | 0.001 | 0.001 | 0.001 | 0.001 | 0.001 |  |
| *Goodness-of-fit* |  |  |  |  |  |  |  |  |
| *AIC* | -1114.513 | -1134.661 | -1137.673 | -1135.674 | -1138.004 | -1136.015 | -1139.710 |  |
| *BIC* | -1103.889 | -1120.496 | -1119.967 | -1114.426 | -1113.215 | -1107.685 | -1107.839 |  |
| *Log Likelihood* | 560.256 | 571.330 | 573.836 | 573.837 | 576.002 | 576.008 | 578.855 |  |
| *^†^ χ^2^* |  | 22.1481 | 5.0120 | 0.0006 | 4.3303 | 0.0114 | 5.6947 |  |
| *^†^ df* |  | 1 | 1 | 1 | 1 | 1 | 1 |  |
| *^†^ p* |  | <.001 | 0.025 | 0.979 | 0.037 | 0.914 | 0.017 |  |
| *For parameters, **p < 0.001, **p < 0.01, *p < 0.05. Observations in each model = 255. Groups (PP) = 17. ^†^The goodness-of-fit test is with respect to the model standing previous in the series of models for the given outcome variable, i.e. Model 2 with respect to Model 1* | | | | | | | | |

# References

- Butz, T. (2006). Fourier transformation for pedestrians. Springer-Verlag, Berlin.
- Ivanov, P. C., Amaral, L. A., Goldberger, A. L., Havlin, S., Rosenblum, M. G., Struzik, Z. R., & Stanley, H. E. (1999). Multifractality in human heartbeat dynamics. *Nature*, *399*(June), 461–465. http://doi.org/10.1038/20924
- Kreuz T, Haas J, Morelli A, Abarbanel HDI, Politi A. Measuring spike train synchrony. *JNeurosci Methods* 165, 151 (2007)
- Kreuz T, Chicharro D, Houghton C, Andrzejak RG, Mormann F: Monitoring spike train synchrony. *JNeurophysio*l 109, 1457 (2013)
- Mandelbrot, B. B. (1983). *The Fractal Geometry of Nature*. New York, NY: W. H. Freeman.
- Muñoz-Diosdado, A. et al. (2005). Some cases of crossover behavior in heart interbeat and electroseismic time series. *Fractals*, 13. doi: 10.1142/S0218348X05002970
- Peng, C.-K., Buldyrev, S. V., Havlin, S., Simons, M., Stanley, H. E., & Goldberger, A. L. (1994). Mosaic organization of DNA nucleotides. *Physical Review E*, *49*(2), 1685–1689. http://doi.org/10.1103/PhysRevE.49.168
